# Multidrivers of energy-related carbon emissions and its decoupling with economic growth in Northwest China

**DOI:** 10.1038/s41598-024-57730-7

**Published:** 2024-03-25

**Authors:** Fang Shen, Abudukeyimu Abulizi

**Affiliations:** 1 College of Geography and Remote Sensing Sciences, Xinjiang University, Urumqi, 830046 China; 2 Xinjiang Key Laboratory of Oasis Ecology, Xinjiang University, Urumqi, 830046 China

**Keywords:** Energy and society, Environmental economics, Sustainability

## Abstract

Northwest China has great natural resource endowment to develop its economy, but factors such as geographic remoteness and technological backwardness result in lower economic levels and higher carbon emissions. This study calculated the energy-related carbon emissions of five provinces in this region, and the evolutionary characteristics of energy-related carbon emissions were analysed from the spatiotemporal perspective. The Kaya identity was applied to decompose the factors influencing energy-related carbon emissions, and the logarithmic mean divisia index (LMDI) and refined Laspeyres index were combined to calculate the role of each influencing factor on energy-related carbon emissions. Finally, the Tapio and LMDI models were used to analyse the evolution of the decoupling relationship between energy-related carbon emissions and economic growth and the role of various influencing factors. The energy-related carbon emissions in Northwest China showed an increasing trend. In terms of influencing factors, economic growth and urban expansion had the highest contributions to carbon emissions and decoupling inhibition, whereas population agglomeration had the opposite effect. Northwest China showed great decoupling trends between energy-related carbon emissions and economic growth.

## Introduction

Owing to its important role in industrial, economic, and social development, energy consumption has been growing rapidly, along with the rapid progress of human civilization. According to the Statistical Review of World Energy published by BP in 2022, global primary energy consumption in 2021 increased by approximately 6% from 2020, suggesting that the considerable decline in carbon emissions in 2020 due to the Coronavirus Disease was only temporary^1^. Global Energy Review 2021 has also stated that a further increase in carbon emissions will accompany a rebound in energy demand by 2021^2^. The International Energy Agency has indicated that the global energy market faces significant pressure in 2021, and 2022 Russia–Ukraine conflict exacerbates energy crisis^3^, and 2023 Israeli-Palestinian conflict disrupts energy transportation. These above data indicate that the mismatch between the rapid growth in energy consumption and the limited energy supply needs to be urgently addressed.

Although energy has contributed greatly to economic growth and social progress^4^, the environmental pollution, increasing greenhouse gas emissions, and resource wastage arising from energy consumption should not be ignored^5^, the most common of which is carbon emissions associated with energy consumption^6^. Energy-related carbon emissions are the product of economic growth, and the interrelationship between the two is not static; their interrelationship not only changes spatiotemporally, but also changes owing to the impact of urbanization^7^, populations^8^, and industries^9^, among other factors. Thus, exploring the interrelationship between the two could foster sustainable economic development, and examining the drivers of this relationship could generate concrete programs for achieving sustainable development at the city, regional, and national levels^10^.

China, as the world’s second largest economy, has experienced rapid economic development in recent times and developed a considerable number of energy industries which have achieved economic growth at the expense of the environment^11^. With the Chinese government promising world carbon neutrality and carbon peaking^12^, how can energy-related carbon emissions be reduced without slowing down economic growth? How can high-quality economic development be achieved in China’s economy? These two issues have become major challenges in China. As the high carbon emission region in China, studying the current situation and driving factors of energy carbon emission in Northwest is conducive to clarifying the carbon reduction direction and practically proposing the carbon reduction path. Determining the decoupling status between economic growth and energy carbon emissions in this region can further judge whether the current development model is reasonable and optimize its economic development concept. By calculating the contribution value of each influencing factor to decoupling, it is possible to further clarify the role of each factor on regional development and propose improvement measures to achieve the desirable decoupling status.

At present, more scholars apply the logarithmic mean divisia index (LMDI)^13^ model to decompose the factors affecting carbon emissions, for example, Ang^14^ uses LMDI model to analyze China's manufacturing carbon emissions, Zhang^15^ employs LEAP model to predict Northwest's carbon peaks under multi-scenarios, Xin^16^ analyses Gansu's carbon emissions via LMDI model, and Jia^17^ studies China's carbon emissions based on LMDI model. Throughout the relevant literature, this study finds that the majority of scholars only using LMDI model to decompose influencing factors. No scholars have yet adopted other methods to verify the calculation accuracy from LMDI model. One of the novelties in this study is to validate the results' accuracy of the LMDI model with those of refined Laspeyres index (RLI)^18^, which proves that the results of these two models are basically same, and there are only minor numerical differences. Most studies solely analyze the role of influencing factors on carbon emissions or in decoupling, and fewer studies combine the two analyses. Another novelty of this study is to analyze both the roles played by the influencing factors on energy carbon emissions and in decoupling, and deeply analyzing the driving role of each influencing factor. There are some deficiencies in this study that need to be further improved. (a) The influencing factors only cover the energy, social and economic dimensions, while policies and technologies should also be considered to make it comprehensive and scientific. (b) This study has not yet identified the causes of the subtle differences between the LMDI and RLI model calculations. (c) This study did not simulate or predict future decoupling evolution.

This study takes Northwest China as the example to investigate the decoupling relationship between energy carbon emissions and economic growth with the help of energy and socio-economic data in 2000–2020.The IPCC method is used to calculate the energy carbon emissions, and the Kaya equation is applied to decompose the influencing factors into seven. The LMDI model and RLI model are employed to validate and compare the contribution value of each influencing factor. The Tapio and LMDI models are combined to calculate the yearly decoupling status and the role of each factor in decoupling.

## Data and methods

### Overview of study area

**Figure 1:**
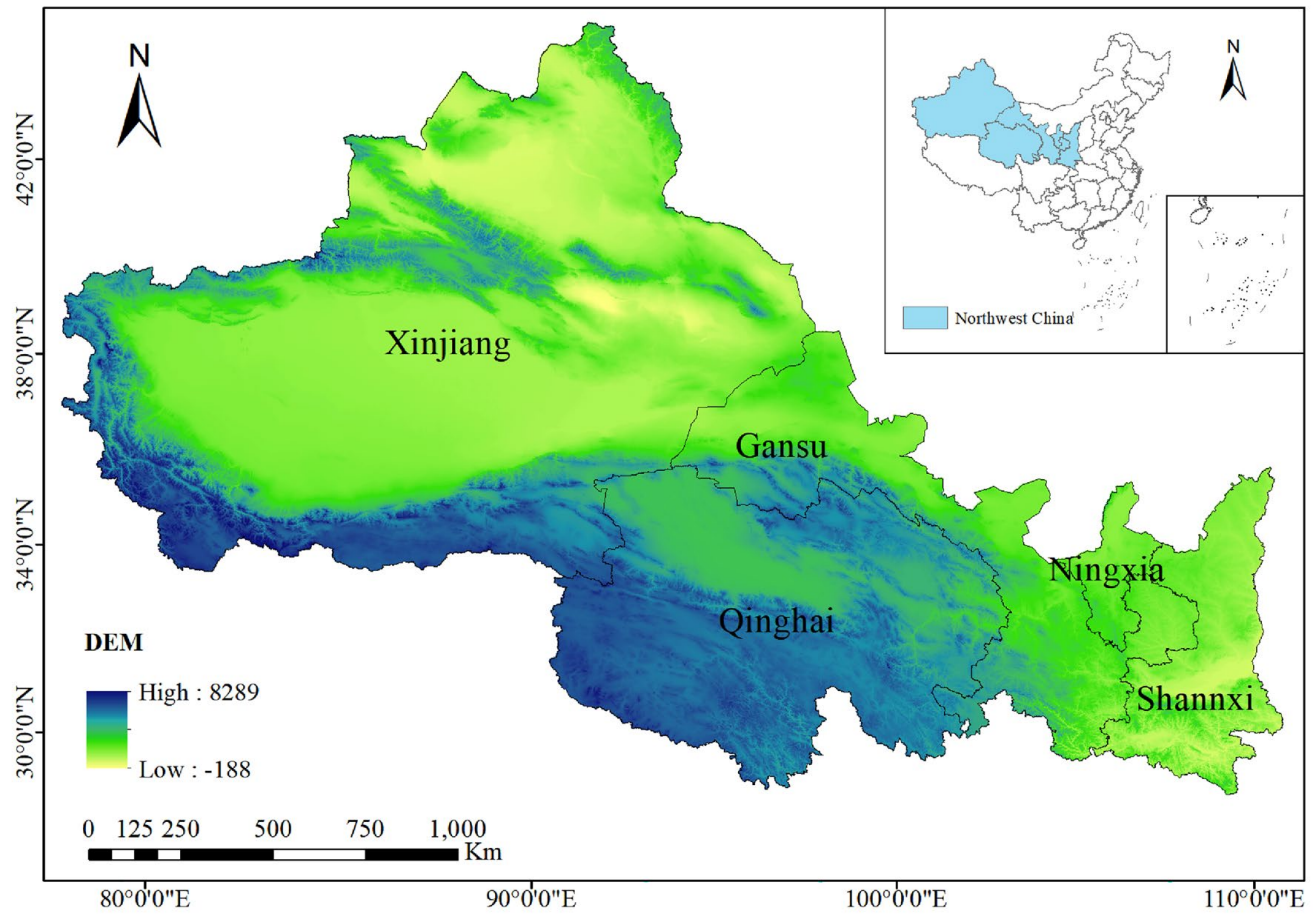
Schematic of study area.

Northwest China (73° 41′–111° 15′ E, 31° 39′–49° 33′ N) is located in the northwestern part of the Chinese territory^19^. According to the administrative division of China, it mainly includes Shaanxi, Gansu, Qinghai, Ningxia, and Xinjiang, and it has an area of approximately 3.09 × 10^6^ km^2^, as shown in Fig. 1. Northwest China is rich in fossil energy, light, and heat resources, and is an important energy base and food production area in China. Along with the implementation of China’s economic development policies such as “Western Development”^20^, “Silk Road Economic Belt”^21^, and “One Belt, One Road”^22^, the utilization of energy resources and the economic development of this region have gradually attracted the attention of Chinese scholars. Although this region is rich in resources and has conditions suitable for sustainable development, it is part of China’s less-developed economy, and experiences serious resource wastage. It is difficult to develop high-tech industries in this region, while the rich resource endowment has intangibly contributed to resource wastefulness^23^. Excessive energy consumption has made this region a high-carbon-emission region within China.

### Data

The energy data used to measure carbon emissions, the socioeconomic energy data used to decompose the seven factors that influence carbon emissions, and the economic data used to calculate decoupling state were obtained from statistical yearbooks for 2000–2020. The data used in this study are official, stable, and reliable. The required data types, sources, and units are listed in Table 1. Some missing data during collection were estimated using the linear interpolation method. The total population data was based on the year-end resident population rather than the year-end total population, the relevant economic data throughout the text were calculated at a constant price for 2000.

**Table 1:** Data types, sources and units.

Section	Data type	Data source	Unit
Energy-related carbon emissions calculation	Coal consumption	Statistical Yearbook	10 thousand Tonnes of standard coal equivalent
	Oil consumption		
	Natural gas consumption		
	Other energy consumption		
	Energy-related carbon emissions	Calculation	Million tonnes of CO_2_ equivalent
Decomposition of influencing factors	Total industrial energy consumption	Statistical yearbook	10 thousand Tonnes of standard coal equivalent
	GDP		0.1 billion yuan
	Industry GDP		
	Year-end resident population		10 thousand
	Built-up area		Square kilometres
	Gross land area		
Calculation of decoupling state	GDP	Statistical Yearbook	0.1 billion yuan
	Energy-related carbon emissions	Calculation	Million tonnes of CO_2_ equivalent

### Methods

#### IPCC method

Currently, the carbon emission calculation methods commonly used in academia include life cycle assessment, input–output analysis, carbon footprint calculations, and the Intergovernmental Panel on Climate Change (IPCC) method^24^. This study chose the IPCC method to calculate energy-related carbon emissions in Northwest China for 2000–2020.

1 $$C^{t} = \mathop \sum \limits_{i}^{4} E_{i} *F_{i} *\frac{44}{{12}}$$

where C^t^ denotes the total energy-related carbon emissions in year t; i from 1 to 4 represents the four energy types of coal, oil, natural gas, and other energy sources used for power generation, respectively; E_i_ denotes the total consumption of energy in category i; and F_i_ denotes the carbon emission coefficient of category i^25^, which was derived from the “Synthesis Report of China’s Analysis of Sustainable Energy and Carbon Emission Scenarios” (Table 2), and 44/12 is the carbon-to-CO_2_ conversion coefficients.

**Table 2:** Energy-related carbon emission coefficients.

Energy type	Coal	Oil	Natural gas	Other energy sources
Carbon emission coefficients (F_i_)	0.7476	0.5825	0.4435	0

#### Kaya identity

Kaya first articulated the Kaya identity in his 1989 article, decomposing the factors influencing energy into energy structure, energy intensity, economic growth, and populations^26^. This study expands on Kaya’s identity by decomposing the factors influencing carbon emissions into seven categories.

2 $$\begin{aligned} C^{t} & = \frac{{C^{t} }}{{E_{I} }}*\frac{{E_{I} }}{{GDP_{I} }}*\frac{{GDP_{I} }}{GDP}*\frac{GDP}{P}*\frac{P}{A}*\frac{A}{U}*U \\ & = ES*EI*SI*G*PA*US*U \\ \end{aligned}$$

where E_I_ denotes the total industry energy consumption; GDP_I_ is the industry GDP; GDP is the total GDP; P is the total year-end resident population; A is the built-up area; U is the total land area (i.e., the land scale). ES is the energy structure, EI is the energy intensity, SI is the industry structure, G is the level of economic growth, PA is population agglomeration, and US is urban sprawl. ES and EI represent energy-level influences; SI and G represent economic-level influences; and PA, US, and U represent social-level influences.

#### LMDI method

This study specifically analysed the role of each decomposition factor on energy-related carbon emissions based on the decomposition results of the Kaya identity using the additive LMDI method.

3 $$\Delta C = C^{t} - C^{0} = \Delta ES + \Delta EI + \Delta SI + \Delta G + \Delta PA + \Delta US + \Delta U$$

where C^0^ denotes total energy-related carbon emissions in base year 0, and ΔC denotes the change in energy-related carbon emissions over period [0,t]. ΔES, ΔEI, ΔSI, ΔG, ΔPA, ΔUS, and ΔU represent the changes in carbon emissions induced by energy structure, energy intensity, industry structure, economic growth, population agglomeration, urban sprawl, and land scale, respectively, from year t to base year 0. The formulas for these variables are as follows:

4 $$\vartriangle ES = \sum \frac{{C^{t} - C^{0} }}{{lnC^{t} - lnC^{0} }}\ln \frac{{ES^{t} }}{{ES^{0} }}$$

5 $$\vartriangle EI = \sum \frac{{C^{t} - C^{0} }}{{lnC^{t} - lnC^{0} }}\ln \frac{{EI^{t} }}{{EI^{0} }}$$

6 $$\vartriangle SI = \sum \frac{{C^{t} - C^{0} }}{{lnC^{t} - lnC^{0} }}\ln \frac{{SI^{t} }}{{SI^{0} }}$$

7 $$\vartriangle G = \sum \frac{{C^{t} - C^{0} }}{{lnC^{t} - lnC^{0} }}\ln \frac{{G^{t} }}{{G^{0} }}$$

8 $$\vartriangle PA = \sum \frac{{C^{t} - C^{0} }}{{lnC^{t} - lnC^{0} }}\ln \frac{{PA^{t} }}{{PA^{0} }}$$

9 $$\vartriangle US = \sum \frac{{C^{t} - C^{0} }}{{lnC^{t} - lnC^{0} }}\ln \frac{{US^{t} }}{{US^{0} }}$$

10 $$\vartriangle U = \sum \frac{{C^{t} - C^{0} }}{{lnC^{t} - lnC^{0} }}\ln \frac{{U^{t} }}{{U^{0} }}$$

Equations (4–10) represents the contributing values of ES, EI, SI, G, PA, US, and U to the energy-related carbon emissions, respectively. Calculation results > 0 will promote carbon emissions, and the opposite will inhibit carbon emissions, while 0 has no effect.

11 $$\eta_{ES} = \frac{\Delta ES}{{\Delta C}}$$

12 $$\eta_{EI} = \frac{\Delta EI}{{\Delta C}}$$

13 $$\eta_{SI} = \frac{\Delta SI}{{\Delta C}}$$

14 $$\eta_{G} = \frac{\Delta G}{{\Delta C}}$$

15 $$\eta_{PA} = \frac{\Delta PA}{{\Delta C}}$$

16 $$\eta_{US} = \frac{\Delta US}{{\Delta C}}$$

17 $$\eta_{U} = \frac{\Delta U}{{\Delta C}}$$

where ΔES, ΔEI, ΔSI, ΔG, ΔPA, ΔUS, and ΔU were calculated as a proportion of ΔC, i.e., the contribution rate of each factor to the energy-related carbon emissions.

#### RLI model

This study computed the contribution values with RLI model and then validated and compared them with the LMDI calculations. Sun assumed that V = x*y^27^, dependent variable V was affected by the x, y factor, and dependent variable V produced change ∆V during [0,t], and the formula for calculating the role played by x, y factor on ∆V was as follows:

18 $$X_{effect} = y^{0} \Delta x + \frac{1}{2}\Delta x\Delta y$$

19 $$Y_{effect} = x^{0} \Delta y + \frac{1}{2}\Delta x\Delta y$$

20 $$\vartriangle V = X_{effect} + Y_{effect}$$

where X_effect_ and Y_effect_ represent the roles played by the x and y factors on ∆V. This study, based on the RLI model, separately calculated the seven factors influencing energy-related carbon emissions using the following formula:

21 $$\begin{aligned} \Delta C & = C^{t} - C^{0} = ES^{t} *EI^{t} *SI^{t} *G^{t} *PA^{t} *US^{t} *U^{t} - ES^{0} *EI^{0} *SI^{0} *G^{0} *PA^{0} *US^{0} *U^{0} \\ & = \left( {ES^{0} + \Delta ES} \right)*\left( {EI^{0} + \Delta EI} \right)*\left( {SI^{0} + \Delta SI} \right)*\left( {G^{0} + \Delta G} \right)*\left( {PA^{0} + \Delta PA} \right)*\left( {US^{0} + \Delta US} \right)*\left( {U^{0} + \Delta U} \right) \\ & \quad - ES^{0} *EI^{0} *SI^{0} *G^{0} *PA^{0} *US^{0} *U^{0} \\ \end{aligned}$$

22 $$\begin{aligned} ES_{effect} & = EI^{0} SI^{0} G^{0} PA^{0} US^{0} U^{0} \Delta ES + \frac{1}{2}\vartriangle ES\left( {EI^{0} SI^{0} G^{0} PA^{0} US^{0} \Delta U + \cdots + \Delta EISI^{0} G^{0} PA^{0} US^{0} U^{0} } \right) \\ & \quad + \frac{1}{3}\Delta ES\left( {\Delta EI\Delta SIG^{0} PA^{0} US^{0} U^{0} + \cdots + EI^{0} SI^{0} G^{0} PA^{0} \Delta US\Delta U} \right) \\ & \quad + \frac{1}{4}\Delta ES\left( {\Delta EI\Delta SI\Delta GPA^{0} US^{0} U^{0} + \cdots + EI^{0} SI^{0} G^{0} \Delta PA\Delta US\Delta U} \right) \\ & \quad + \frac{1}{5}\Delta ES\left( {\Delta EI\Delta SI\Delta G\Delta PAUS^{0} U^{0} + \cdots + EI^{0} SI^{0} \Delta G\Delta PA\Delta US\Delta U} \right) \\ & \quad + \frac{1}{6}\Delta ES\left( {\Delta EI\Delta SI\Delta G\Delta PA\Delta USU^{0} + \cdots + EI^{0} \Delta SI\Delta G\Delta PA\Delta US\Delta U} \right) \\ & \quad + \frac{1}{7}\Delta ES\Delta EI\Delta SI\Delta G\Delta PA\Delta US\Delta U \\ \end{aligned}$$

23 $$\vartriangle C = ES_{effect} + EI_{effect} + SI_{effect} + G_{effect} + PA_{effect} + US_{effect} + U_{effect}$$

Equation (22) is the ES contribution value to energy-related carbon emissions, and it should be noted that, owing to the complexity of the formula for calculating the influencing factors by the RLI, this study only presents the formula for calculating the ES contribution value in this context.

24 $$\Delta C_{ES} = \frac{{ES_{effect} }}{\Delta C}$$

25 $$\Delta C_{EI} = \frac{{EI_{effect} }}{\Delta C}$$

26 $$\Delta C_{SI} = \frac{{SI_{effect} }}{\Delta C}$$

27 $$\Delta C_{G} = \frac{{G_{effect} }}{\Delta C}$$

28 $$\Delta C_{PA} = \frac{{PA_{effect} }}{\Delta C}$$

29 $$\Delta C_{US} = \frac{{US_{effect} }}{\Delta C}$$

30 $$\Delta C_{U} = \frac{{U_{effect} }}{\Delta C}$$

where ES_effect_, EI_effect_, SI_effec_, G_effect,_ PA_effect_, US_effect_, and U_effect_ represent the contribution values of the seven influencing factors to energy-related carbon emissions, and ΔC_ES_, ΔC_EI_, ΔC_SI_, ΔC_G_, ΔC_PA_, ΔC_US_, and ΔC_U_ represent the contribution rate of the seven influencing factors to the energy-related carbon emissions, respectively.

#### Tapio decoupling model

**Figure 2:**
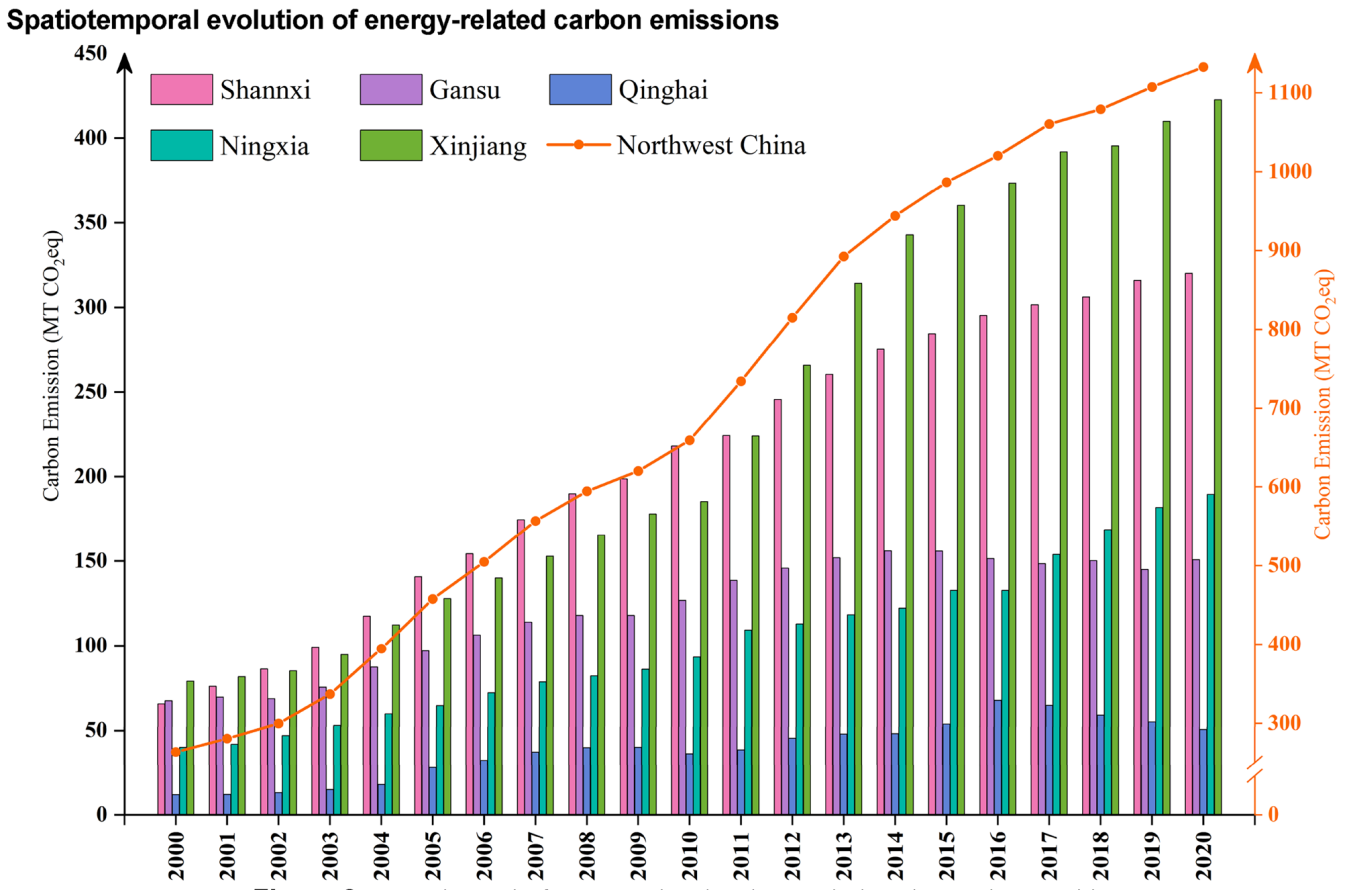
Growth trend of energy-related carbon emissions in Northwest China.

This study used the decoupling model proposed by Tapio in 2005^28^, which is calculated as follows:

31 $$\alpha_{t} = \frac{{\Delta C/C^{0} }}{{\Delta GDP/GDP^{0} }}$$

where α_t_ denotes the decoupling elasticity coefficient in year t; GDP^0^ denote GDP in the base year 0; and ΔGDP denote the amount of change in GDP during [0,t], respectively. Based on the values of α_t_, ΔC, and ΔGDP, eight decoupling states are shown in Fig. 2. Referring to Eqs. (3–10), this study further expressed the decoupling model as follows:

32 $$\alpha_{t} = \frac{{\left( {\Delta ES + \Delta EI + \Delta SI + \Delta G + \Delta PA + \Delta US + \Delta U} \right)/C^{0} }}{{\Delta GDP/GDP^{0} }}$$

33 $$\alpha_{t} = \Delta ES\frac{{GDP^{0} }}{{C^{0} \Delta GDP}} + \Delta EI\frac{{GDP^{0} }}{{C^{0} \Delta GDP}} + \Delta SI\frac{{GDP^{0} }}{{C^{0} \Delta GDP}} + \Delta G\frac{{GDP^{0} }}{{C^{0} \Delta GDP}} + \Delta PA\frac{{GDP^{0} }}{{C^{0} \Delta GDP}} + \Delta US\frac{{GDP^{0} }}{{C^{0} \Delta GDP}} + \Delta U\frac{{GDP^{0} }}{{C^{0} \Delta GDP}}$$

34 $$\alpha_{t} = \alpha_{ES} + \alpha_{EI} + \alpha_{SI} + \alpha_{G} + \alpha_{PA} + \alpha_{US} + \alpha_{U}$$

where α_ES_, α_EI_, α_SI_, α_G_, α_PA_, α_US_, and α_U_ denote the contribution value of each influencing factor to the decoupling elasticity index α in year t. The values of the factors contributing to the decoupling state were quantified using the formulas above. Table 3 describe eight decoupling states, which are arranged in order of ideality: strong decoupling (SD) > weak decoupling (WD) > expansive coupling (EC) > expansive negative decoupling (END) > recessive decoupling (RD) > recessive coupling (RC) > weak negative decoupling (WND) > strong negative decoupling (SND).

**Table 3:** Classification of decoupling state.

ΔGDP	ΔC	α_t_	Decoupling state	State assessment
ΔGDP > 0	ΔC < 0	α_t_ < 0	Strong decoupling (SD)	Optimal; the economy grows, while energy-related carbon emissions decline
ΔGDP > 0	ΔC > 0	0 ≤ α_t_ < 0.8	Weak decoupling (WD)	Very good; the growth rate of the economy is faster than that of energy-related carbon emissions
ΔGDP < 0	ΔC < 0	α_t_ > 1.2	Recessive decoupling (RD)	Average; the recession rate of the economy is slower than that of energy-related carbon emissions
ΔGDP < 0	ΔC < 0	0 ≤ α_t_ < 0.8	Weak negative decoupling (WND)	Very poor; the recession rate of the economy is faster than that of energy-related carbon emissions
ΔGDP < 0	ΔC > 0	α_t_ < 0	Strong negative decoupling (SND)	Extremely poor; energy-related carbon emissions increases, while the economy declines
ΔGDP > 0	ΔC > 0	α_t_ > 1.2	Expansive negative decoupling (END)	Relatively good; the growth rate of the economy is slower than that of energy-related carbon emissions
ΔGDP > 0	ΔC > 0	0.8 ≤ α_t_ ≤ 1.2	Expansive coupling (EC)	Good; the growth rate of economy is basically equal to that of energy-related carbon emissions
ΔGDP < 0	ΔC < 0	0.8 ≤ α_t_ ≤ 1.2	Recessive coupling (RC)	Poor; the recession rate of economy is basically equal to that of energy-related carbon emissions

## Results

### Spatiotemporal evolution of energy-related carbon emissions

**Figure 3:**
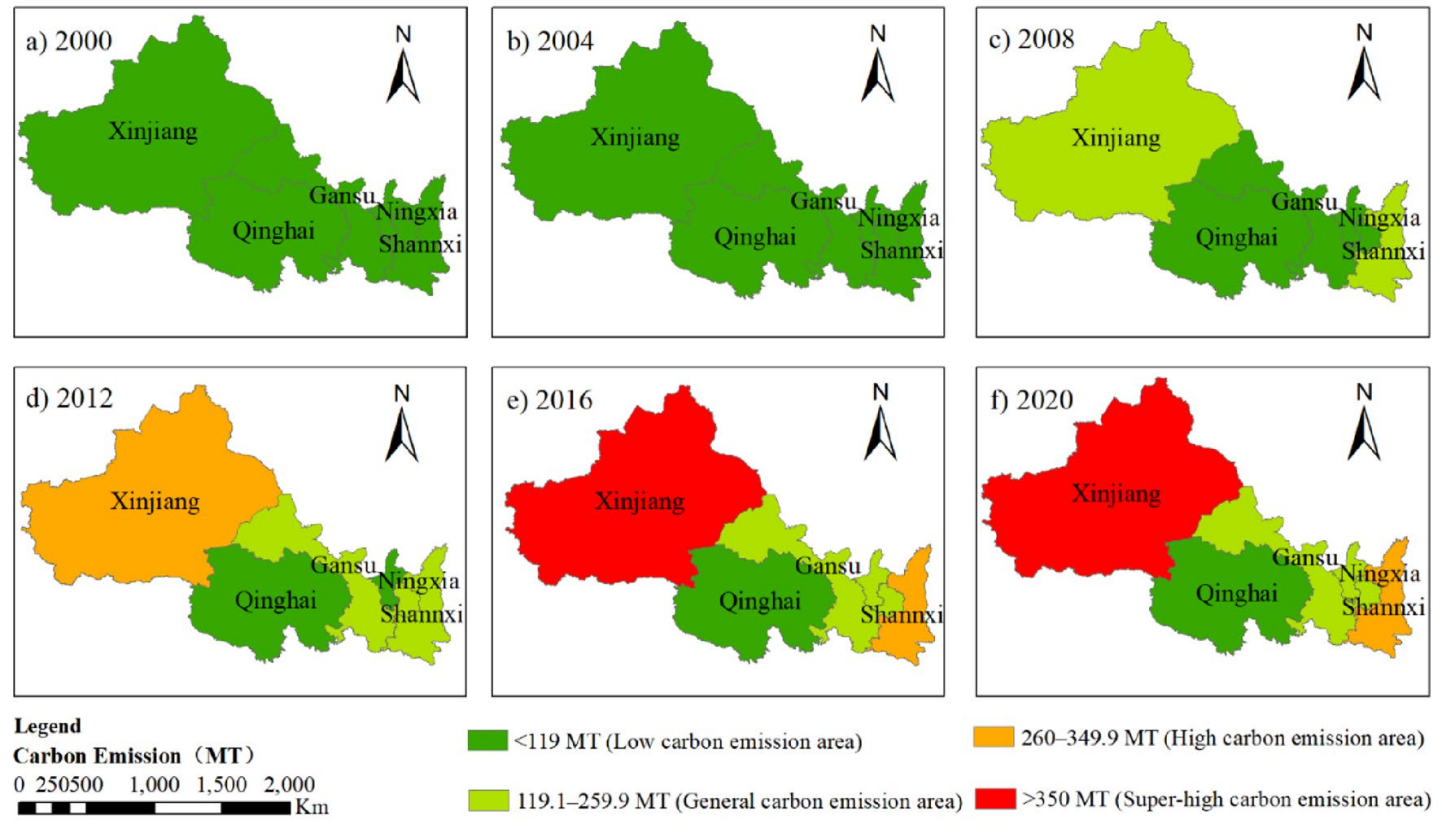
Spatiotemporal changes in energy-related carbon emissions of Northwest in 2000, 2004, 2008, 2012, 2016, and 2020.

As shown in Fig. 2, energy-related carbon emissions in Northwest China increased from 264 MT CO_2_eq in 2000 to 1,133 MT CO_2_eq in 2020, an overall increase of 329.17%. This study categorised the five provinces in Northwest China with reference to carbon emissions, which are mainly divided into super-high (> 350 MT), high (260–349.9 MT), general (119.1–259.9 MT), and low carbon emission area (< 119 MT). The main comparison was the spatiotemporal changes in energy-related carbon emissions in 2000, 2004, 2008, 2012, 2016, and 2020 (Fig. 3).

Overall, Shannxi, Gansu, Qinghai, Ningxia, and Xinjiang showed steady growth trends. Qinghai has always been the province with the lowest carbon emissions and has remained a low-carbon emission area, with the second lowest average annual growth rate of carbon emissions, which is the result of the dual role of its level of economic development and reasonable energy structure^29^. Shannxi had the highest carbon emissions in 2002–2011 but was then overtaken by Xinjiang, which eventually became a high-carbon emission area. The main reason is that Shannxi was listed as a national low-carbon pilot province in 2012, as it practically adjusted its energy consumption structure and improved its energy intensity^30^. In addition, Shannxi actively responded to the national “13th Five-Year Plan for Energy Development” in 2017 by “subtracting” energy conservation and “adding” economic development^31^. Despite the implementation of aggressive low-carbon emission policies after 2012, Shannxi still maintained high carbon emissions as its economy continued to expand. Xinjiang has the highest average annual growth rate of carbon emissions in Northwest China, at 21.76%, and was the first to enter the general carbon emission area in 2008, becoming a super-high carbon emission area in 2016 and 2020. The cold and lingering winter climate of Xinjiang causes winter heating to consume huge amounts of energy^32^, and the sparse population makes Xinjiang’s transportation sector consume huge amounts of fuel. Xinjiang maintained high carbon emissions, which was caused by three aspects: society, climate, and policy. Gansu had the lowest annual carbon emission growth rate, 6.18%, mainly because of its abundant clean energy and increasing share of clean energy consumption^33^. Ningxia ranked second lowest in carbon emissions until 2016, after which it was overtaken by Shannxi. Although both Gansu and Ningxia transitioned from being low-to high-carbon emission areas, the transition speed of Ningxia was slower than that of Gansu. Because Ningxia’s annual carbon emission growth rate was higher than that of Gansu, Ningxia’s carbon emission base was smaller.

### Decomposition analysis of energy-related carbon emission drivers

**Figure 4:**
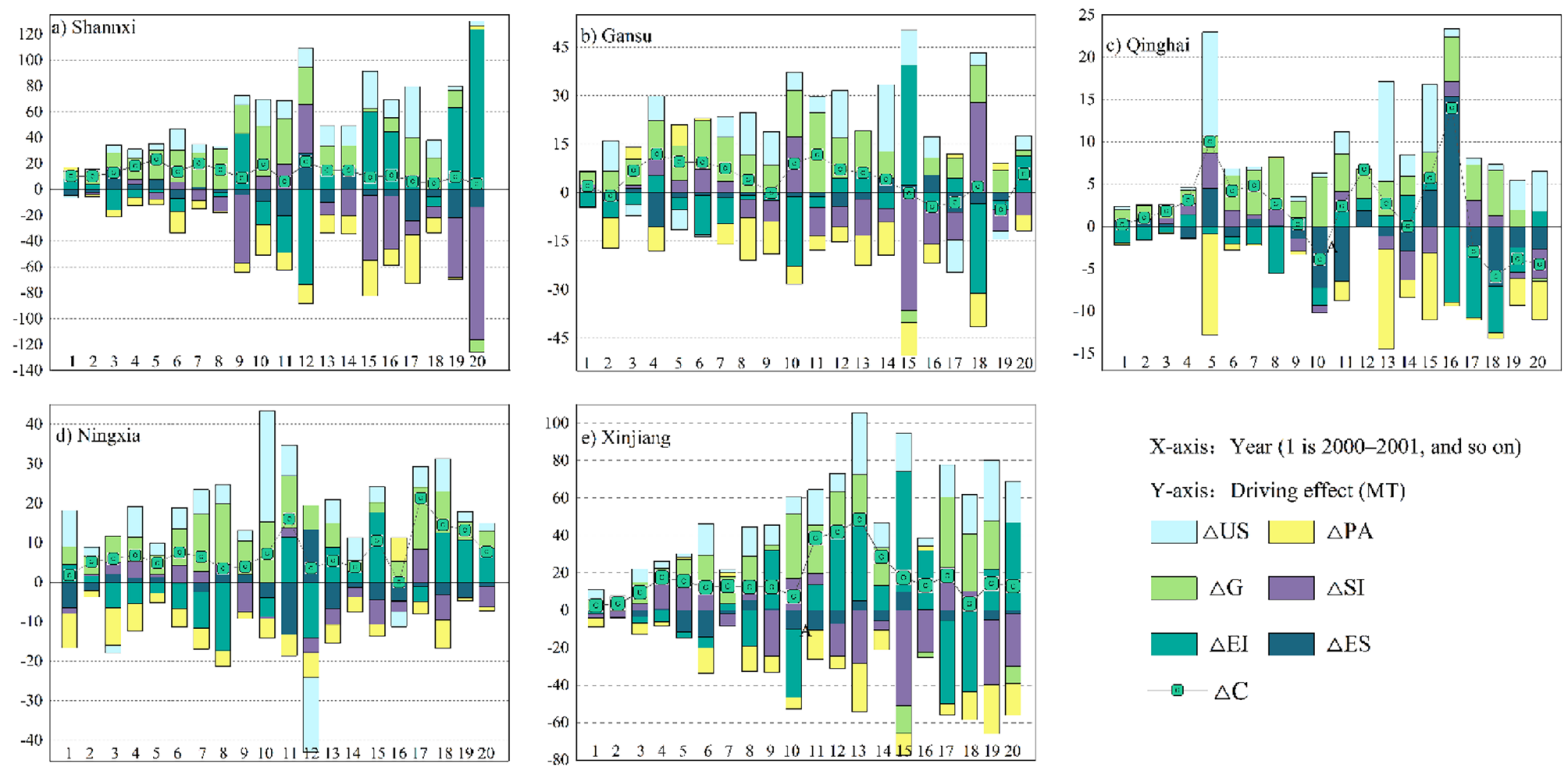
Contribution values of energy-related carbon emission drivers.

The contribution values of the influencing factors of energy-related carbon emissions in Shannxi, Gansu, Qinghai, Ningxia, and Xinjiang from 2000 to 2020 were calculated using Eqs. (4–10) (Fig. 4). Based on Fig. 4, the influencing factors within the provinces varied over time. It is worth noting that U often had a 0 value, and some years with non-0 values had a minor impact on carbon emissions. Therefore, this section does not explain the U influencing factor in detail.

In Shannxi, G and US steadily contributed to the growth of carbon emissions, with G contributing more than US in most years. PA steadily and consistently inhibited the growth of carbon emissions, while the effects of ES, EI, and SI were unstable with large variations over time; however, ES and SI inhibited carbon emissions in more years than they promoted them. G’s contribution to carbon emissions continued to increase in 2000–2011 but showed a fluctuating decline in 2011–2016. Shannxi focused on the speed of economic development before 2011 and then gradually adjusted the economic structure and changed the mode of economic development^34^, which also verifies that SI and ES gradually strengthened the inhibition effect on carbon emissions after 2011. The abundant coal resources and low coal prices have prompted the Shannxi industry to choose coal as its primary energy source, and the irrational energy structure and fluctuating industrial output have rendered the role of EI unstable.

The variations in the influencing factors within Gansu were relatively heterogeneous, with ES, EI, and PA inhibiting carbon emissions and SI, G, and US promoting carbon emissions. In terms of contribution rate, the SI had the highest positive contribution to carbon emissions, at 1753.36%, and US had the highest negative contribution to carbon emissions, at 1195.01%.

According to the average value of each contributing factor, ES, SI, and PA inhibited carbon emissions in Qinghai, and the intensity of inhibition followed the order of SI > ES > PA. EI, G, and US promoted carbon emissions in Qinghai, and the ranking of their contributions followed the order of EI > G > US. However, the role of each factor on carbon emissions fluctuated greatly each year; PA was quite stable and inhibited carbon emissions, while G and US were quite stable and promoted carbon emissions. Overall, the inhibitory effect of ES on carbon emissions was related to the continuing increase in the share of clean energy consumption in Qinghai. G and US contributed to Qinghai carbon emissions during 2000–2016 and inhibit them from 2017 onwards, whereas the PA situation was the exact opposite. This was mainly because, after 2016, Qinghai responded to the national two-child policy and the “13th Five-Year Plan for Population Development in Qinghai”^35^, and the population growth rate was higher than the rate of built-up area expansion and economic development.

PA steadily inhibited Ningxia’s carbon emissions; ES repeatedly alternated between inhibition and promotion during 2000–2020; G and US promoted carbon emissions, except for occasional inhibition; and EI and SI were more repetitive. Ningxia has responded positively to the national economic development policy and has carried out urban development and construction in a steady manner; thus, G and US have contributed to carbon emissions from Ningxia. In 2003, 2008, and 2012, ES went from inhibiting to promoting carbon emissions, which was mainly because the fossil energy consumption share of Ningxia’s industrial sector in the above years was higher than that of the previous year. In 2012, Ningxia actively conformed to the development direction of the 18th Party Congress, while Ningxia tended to rationalize the industrial structure^36^, and SI mainly inhibited carbon emissions.

According to the average contribution rate, ES, EI, SI, and PA mainly inhibited Xinjiang’s carbon emissions, with the inhibition effect being in the order of PA > EI > ES > SI, while G and US mainly promoted carbon emissions in Xinjiang, and the contribution rate of G was larger than that of US. The increased downward economic pressure in 2015, 2016, and 2020 caused Xinjiang’s GDP growth rate to be lower than the population growth rate; thus, G inhibited carbon emissions in these 3 years. Guided by the Xinjiang government’s proactive and robust development policies, US has steadily contributed to the growth of carbon emissions. Because an increase in population agglomeration can considerably reduce people’s transportation energy consumption and heating energy consumption, PA has the most evident inhibiting effect on carbon emissions in Xinjiang. Since Xinjiang eliminated backward industries and upgraded the local industrial structure in 2010, ES and SI more frequently inhibited carbon emissions.

### Validation and comparison of energy-related carbon emission drivers

**Figure 5:**
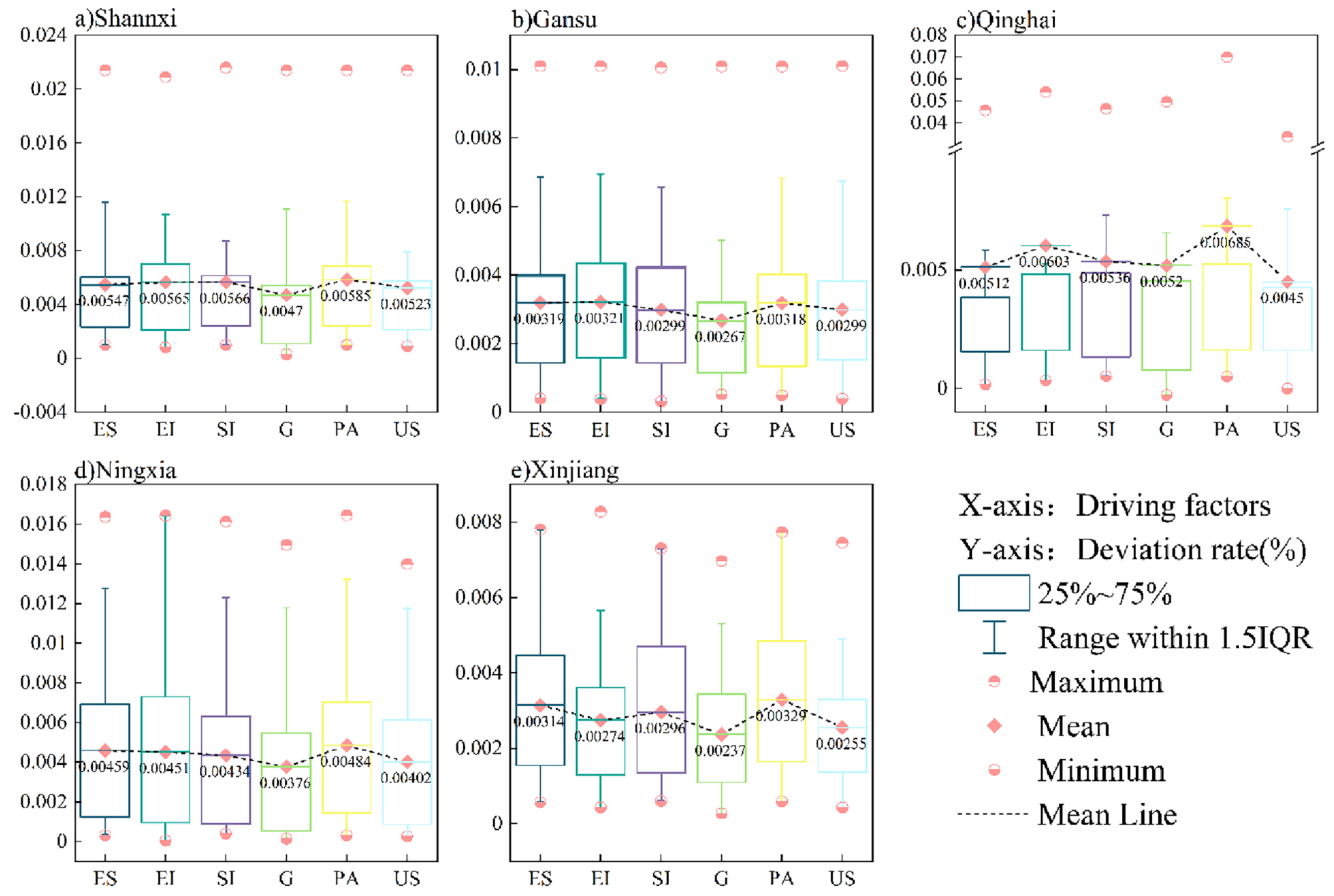
Differences between LMDI and RLI calculation results.

**Figure 6:**
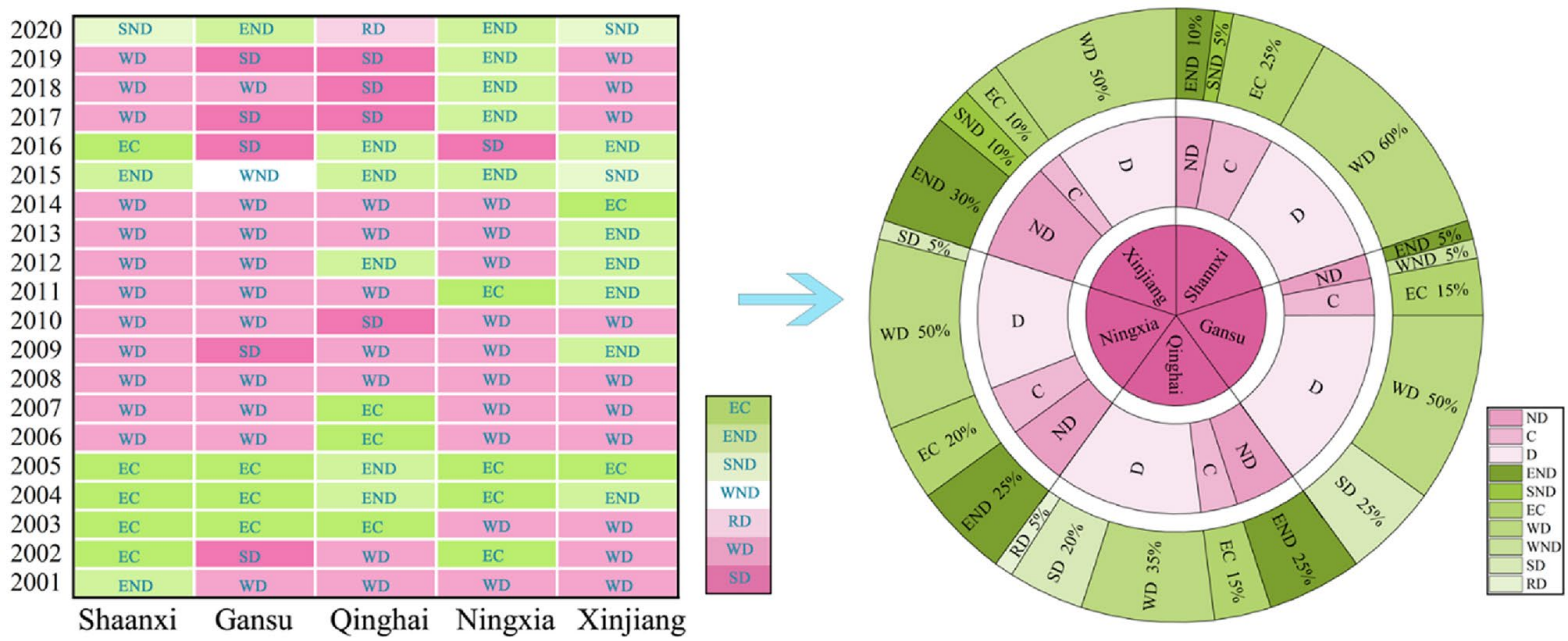
Statistical chart of changes in decoupling state.

Table 4 presents the calculation results of Eqs. (4–10) and (23). Owing to context limitations, this study only compared Shannxi’s contribution values based on the two models in the main text. Table 4 shows that the contribution values of the influencing factors calculated by LMDI and RLI models were basically the same. In terms of the absolute values, the results of the RLI model calculations were slightly larger than those of the LMDI model, but the differences in the absolute values were small. To uniformly show the differences by two calculation methods in Shannxi, Gansu, Qinghai, Ningxia, and Xinjiang during 2000–2020, this study mainly used the RLI model calculation results to subtract those of the LMDI model and then divided them by the RLI model calculation results, which are shown in Fig. 5. Notably, the U influencing factor often has a 0 value. Therefore, Fig. 6 mainly shows the differences in ES, EI, SI, G, PA, and US.

**Table 4:** Comparison of the contribution value of energy- related carbon emission influencing factors in Shannxi based on LMDI and RLI models.

Year	ES		EI		SI		G		PA		US		U	
	LMDI	RLI	LMDI	RLI	LMDI	RLI	LMDI	RLI	LMDI	RLI	LMDI	RLI	LMDI	RLI
2001	− 0.45	− 0.45	0.80	0.80	− 0.03	− 0.03	0.57	0.57	0.25	0.25	− 0.15	− 0.15	0.01	0.01
2002	− 0.23	− 0.23	0.40	0.40	− 0.13	− 0.13	0.93	0.93	− 0.15	− 0.15	0.18	0.18	0.00	0.00
2003	1.13	1.13	− 1.23	− 1.25	0.20	0.21	0.87	0.88	− 0.41	− 0.42	0.44	0.45	0.00	0.00
2004	0.20	0.20	− 0.34	− 0.34	0.22	0.22	0.90	0.90	− 0.35	− 0.35	0.38	0.38	− 0.01	− 0.01
2005	0.34	0.34	− 0.11	− 0.11	− 0.21	− 0.21	0.96	0.96	− 0.18	− 0.18	0.20	0.21	0.00	0.00
2006	− 0.50	− 0.50	− 0.79	− 0.79	0.44	0.44	1.81	1.81	− 1.18	− 1.18	1.21	1.21	0.01	0.01
2007	0.10	0.10	− 0.02	− 0.02	− 0.41	− 0.41	1.31	1.31	− 0.28	− 0.29	0.31	0.31	0.00	0.00
2008	− 0.14	− 0.14	− 0.23	− 0.23	− 0.74	− 0.74	2.07	2.07	− 0.07	− 0.07	0.12	0.12	0.00	0.00
2009	− 0.48	− 0.49	4.89	4.95	− 5.93	− 6.01	2.46	2.49	− 0.78	− 0.79	0.84	0.85	0.00	0.00
2010	− 0.47	− 0.48	− 0.94	− 0.95	0.53	0.53	2.00	2.01	− 1.20	− 1.21	1.09	1.10	0.00	0.00
2011	− 3.23	− 3.24	− 4.60	− 4.63	3.17	3.19	5.57	5.59	− 2.19	− 2.20	2.28	2.29	0.00	0.00
2012	1.31	1.33	− 3.42	− 3.47	1.75	1.77	1.33	1.34	− 0.68	− 0.69	0.71	0.72	0.00	0.00
2013	− 0.69	− 0.70	0.76	0.76	− 0.64	− 0.64	1.52	1.52	− 0.94	− 0.94	1.02	1.02	− 0.03	− 0.03
2014	0.87	0.87	0.22	0.22	− 1.36	− 1.36	1.22	1.22	− 0.96	− 0.96	1.02	1.02	0.00	0.00
2015	− 0.50	− 0.51	6.44	6.49	− 5.36	− 5.41	0.29	0.29	− 2.97	− 3.00	3.11	3.14	0.00	0.00
2016	− 0.46	− 0.47	4.09	4.10	− 3.75	− 3.76	0.99	0.99	− 1.17	− 1.17	1.31	1.31	0.00	0.00
2017	− 3.86	− 3.88	1.65	1.66	− 1.73	− 1.74	4.65	4.67	− 6.00	− 6.03	6.28	6.31	0.00	0.00
2018	− 1.14	− 1.14	− 1.70	− 1.70	− 1.92	− 1.93	5.27	5.27	− 2.44	− 2.44	2.93	2.93	0.00	0.00
2019	− 2.28	− 2.29	6.54	6.57	− 4.73	− 4.76	1.37	1.38	− 0.18	− 0.18	0.27	0.27	0.00	0.00
2020	− 3.29	− 3.36	29.74	30.37	− 24.70	− 25.25	− 2.24	− 2.29	0.67	0.69	0.82	0.84	0.00	0.00

In each subplot in Fig. 5, the pink circles represent the maximum and minimum values of the differences. The pink diamonds represent the mean values of the differences and the black lines represent the mean connecting lines of the differences between ES, EI, SI, G P, A, and US. The lower and upper lines of the rectangular box represent the 25% and 75% quartiles, respectively. The differences between the drivers were small, and the deviation rates were generally positive and remained at approximately 0.5%. Overall, the deviation rates of Gansu and Xinjiang were lower than those of the other three provinces, whereas the deviation rate of Qinghai was higher. Figure 5 and Table 4 show that the deviation rates of G and US were smaller than those of the other factors, which may because economic development and built-up area construction, as an important part of government control, are not only affected by national policies but also comply with the local government’s development plan. Therefore, the trends of G and US were more stable. The ES, EI, SI, and PA deviation rates varied considerably within provinces, mainly because of the different development conditions within provinces and large variations within the study period.

The two decomposition methods above produced no residual terms, and the driving effects of the influencing factors were consistent, with only minor deviations in the values. Both passed the test of factor reversal and time reversal, which can be applied for conducting factor decomposition analysis. Therefore, using the LMDI model to decompose the driving effect of energy-related carbon emissions from energy sources is scientific and reasonable, and the results can provide a reference and support for future research.

### Analysis of the decoupling between energy-related carbon emissions and economic development

This section examines both the evolution of decoupling state and analyses the drivers of decoupling state. The decoupling elasticity indices of Shannxi, Gansu, Qinghai, Ningxia, and Xinjiang were calculated in 2000–2020 using Eq. (32). According to Eqs. (33–35), the contribution value of each influencing factor was calculated, and the effects of the influencing factors were specifically analysed.

#### Evolution of decoupling state

Based on the calculation obtained by Eq. (32), the decoupling state of energy-related carbon emissions and economic growth in Shannxi, Gansu, Qinghai, Ningxia, and Xinjiang during 2000–2020 are shown in Fig. 6, respectively. As shown in Fig. 6, four decoupling states (WD, EC, END, and SND) appeared in Shannxi during 2000–2020, of which WD accounted for the largest proportion, mostly appearing in 2006–2014 and 2017–2019, as the economy grows faster than carbon emissions. Shannxi’s economic growth slowed in 2015 and 2016 when it was seeking a new economic direction, thus presenting END and EC, respectively. Shannxi relied more on the industrial economy, with coal as the major energy source in 2002–2005, thus presenting EC, where the growth rate of economy is equal to that of energy-related carbon emissions. Shannxi was affected by the Coronavirus Disease in 2020, showing SND.

In Gansu, there were five decoupling states in 2000–2020: SD, WD, EC, END, and WND, with WD accounting for the largest share, up to 50%, and SD (25%) was higher than EC (15%), END, and WND, each accounting for 5%. Overall, Gansu’s decoupling trend has developed well. Gansu actively responded to China’s Western Development Program since 2006 and continuously optimized the energy consumption structure since 2004, presenting optimal SD in 2016, 2017, and 2019. The economic recession of WND occurred in 2015, when the severe and complex external environmental and considerable downward pressures were faced by the economy.

**Figure 7:**
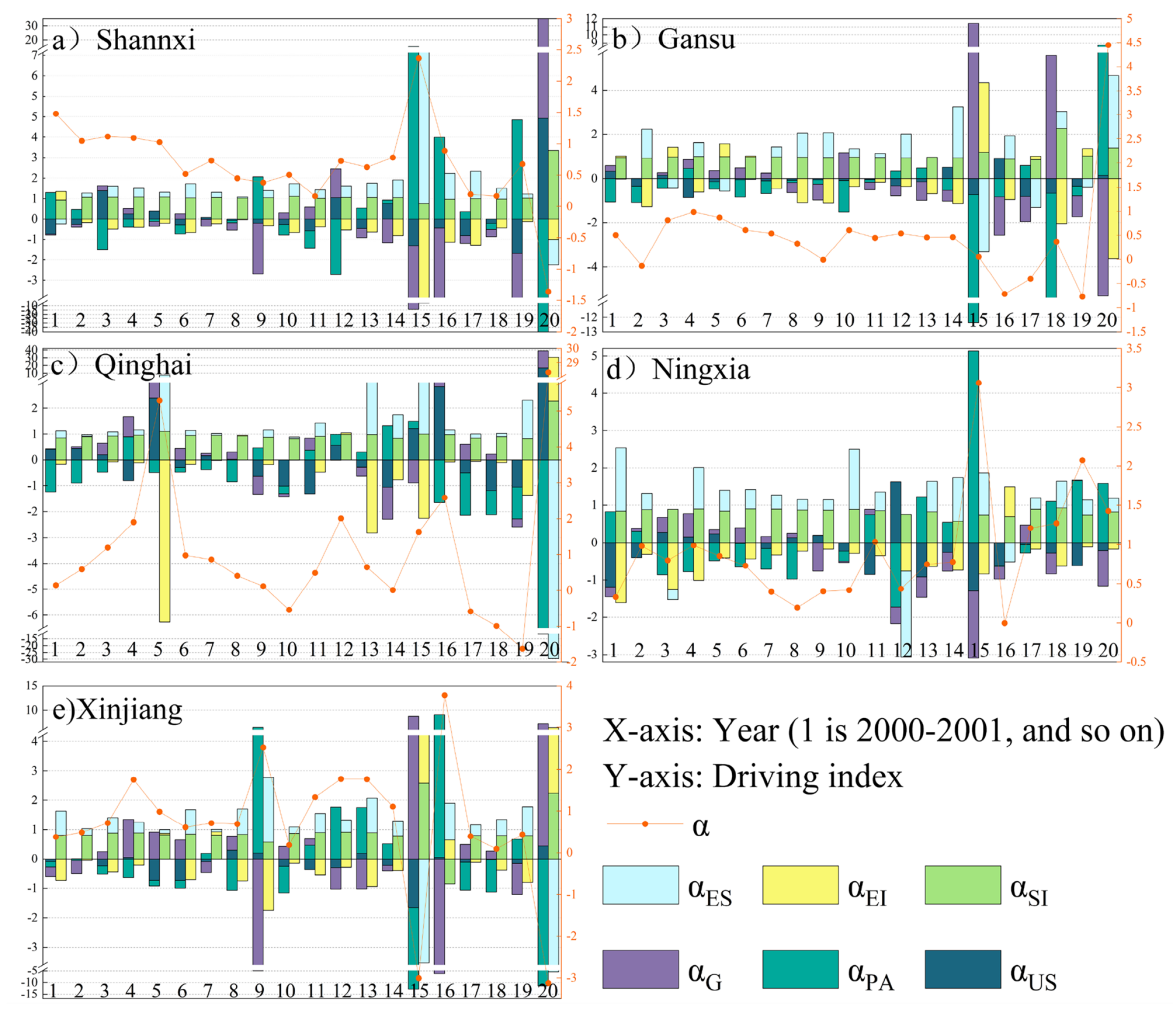
Diagram of decoupling state drivers.

Five decoupling states occurred in Qinghai from 2000 to 2020: WD (35%) > END (25%) > SD (20%) > EC (15%) > RD (5%), in descending order of the rate of each state. It is clear from Fig. 7 that the decoupling state distribution in Qinghai was dispersed; however, overall, it shows a benign decoupling trend. Qinghai actively implemented various energy and economic policies during 2003–2016, resulting in the alternation of WD, END, and EC. Qinghai maintained an economic growth rate above 4% except for 2020, when it was affected by the Coronavirus Disease, presenting RD. Accompanied by an increasingly rationalised energy consumption structure, it showed SD in 2010 and 2017–2019.

There were four decoupling states in Ningxia in 2000–2020: WD (50%) > END (25%) > EC (20%) > SD (5%), in descending order of the rate of each state. Along with the general background of China’s rapid economic development in the early period, the annual growth rate of Ningxia’s economy before 2014 exceeded 4%, which is higher than the growth rate of carbon emissions; thus, WD was mostly distributed between 2001 and 2014. For the first time, Ningxia’s share of clean energy consumption increased to 15% in 2016; hence, SD appeared in 2016. With the slowdown in economic growth since 2017, END was presented in 2017–2020.

There were four decoupling states in Xinjiang for 2000–2020: WD (50%) > END (30%) > EC (10%) = SND (10%), in descending order of the rate of each state. The proportion of decoupling states in Xinjiang was similar to that in Ningxia, but the overall temporal ranking of the decoupling states was quite different. Economic growth was faster than carbon emission increases in 2001–2003, 2006–2008, and 2017–2019, with WD being concentrated during these periods. The distribution of END was relatively scattered, showing a higher concentration in 2011–2013. Xinjiang was affected by downward economic pressure in 2015 and the Coronavirus Disease in 2020, thus presenting the least ideal SND in these 2 years.

From Fig. 6, although the optimal SD did not dominate, the favorable decoupling states, such as WD, EC, and END, accounted for a large percentage, up to more than 75%. Thus, energy-related carbon emissions and economic development in Northwest China showed great decoupling trend, with WD being presented in most years.

#### Analysis of the contribution of decoupling state drivers

Based on the calculations by Eqs. (33–35), the contribution values of Shannxi, Gansu, Qinghai, Ningxia, and Xinjiang decoupling state drivers in 2000–2020 are shown in Fig. 7. Considering that economic regression rarely occurred during the study period, combined with the analysis results before, seven decoupling states appeared in Northwest China, with RD, WND, and SND being presented fewer times and WD, SD, END, and EC being presented more frequently.

The subplots of Fig. 7 represent the decomposition of decoupling state within each province, the pink triangles represent the decoupling elasticity index for each year, and shows the ES, EI, SI, G, PA and US effects on the decoupling state; U was omitted because it often appears as 0. Figure 7a shows the decomposition of Shannxi’s decoupling elasticity index, from which is clear that Shannxi’s decoupling elasticity index showed small fluctuations in 2000–2014, concentrating on [0,2] and all positive values, and large fluctuations in 2015–2020, with a negative value in 2020. Based on the contribution values of influencing factors, G and US were fairly consistent in hindering decoupling, whereas PA remained stable in promoting decoupling. ES and SI more frequently inhibited decoupling, and EI was likely to inhibit decoupling, since Shannxi responded to the low-carbon policy in 2012.

Figure 7b shows Gansu’s decoupling elasticity index decomposition, where G consistently inhibits decoupling, ES and EI promote decoupling more frequently, PA and US have recurrent effects on decoupling, and SI consistently inhibits decoupling in 2000–2007 and then promotes decoupling. ES will continue to promote its development towards an ideal decoupling state, along with the ongoing exploitation of wind and light energy by Gansu government. Gansu rationalised its industry structure since 2008, after which SI promoted decoupling.

Figure 7c shows the decomposition of the Qinghai decoupling elasticity index, with G persistently inhibiting decoupling. US continued to inhibit decoupling, while PA promoted it, except in 2020. SI inhibited decoupling relatively frequently, whereas ES and EI acted more recurrently on decoupling. PA and US were anomalous in 2020 because of the economic recession caused by the Coronavirus Disease. Qinghai experienced repeated energy structure optimisation, which in turn led to repeated decoupling effects of ES.

Figure 7d shows Ningxia’s decoupling elasticity index decomposition, with G and US consistently inhibiting decoupling; PA consistently promoting decoupling; and ES, EI, and SI playing more recurring roles in decoupling. The traditional industrial sector in Ningxia actively improved fossil energy utilization in 2003–2008; thus, EI promoted decoupling during this period. Ningxia has been on the energy structure optimisation path since 2012, forming a stable optimisation trend. Therefore, ES contributed to decoupling after 2012.

Figure 7e shows the decoupling elasticity index decomposition in Xinjiang, with US consistently inhibiting decoupling; G inhibiting decoupling except in 2015, 2016, and 2020; PA contributing to decoupling rather often; and ES, EI, and SI acting more repetitively on decoupling. G promoted decoupling in 2015 and 2016 due to downward economic pressure and in 2020 due to the Coronavirus Disease. Xinjiang’s population was too aggregated to achieve aggregation-reducing carbon emissions effects in 2005, 2007, and 2016; therefore, PA inhibited decoupling in these 3 years.

## Discussion and conclusions

This study focused on the drivers of energy-related carbon emissions and its decoupling with economic growth, and it explored the drivers of decoupling. First, the IPCC method was used to measure energy-related carbon emissions. Second, the LMDI and RLI models were used to calculate and validate the contribution of each influencing factor of energy-related carbon emissions. Finally, the Tapio and LMDI models were used to analyse decoupling states and the effects of each factor on decoupling. The main conclusions are as follows.

1. Energy-related carbon emissions in Northwest China are generally showing a rising trend, with a gradual slowdown in the growth rate and negative growth in certain years. Energy consumption in the Northwest is closely linked to its economic development, and carbon emissions have naturally increased steadily along with its positive and sound economic development. However, it has reduced carbon emissions by increasing the share of clean energy consumption, improving the fossil energy utilization rate, and adjusting the industrial structure, which has slowed down carbon emission growth gradually.
2. Overall, G and US contribute to carbon emissions in Northwest, PA inversely, with ES, EI, and SI playing an inconsistent role. Since economic growth and urban expansion inherently develop themselves by consuming energy, G and US promote carbon emissions. Increased population agglomeration reduces energy consumption significantly in transportation, lighting, heating, etc., so PA suppresses carbon emissions. Optimization of energy structure, improvement of energy use intensity and rationalization of industrial structure could reduce carbon emissions, however, the Northwest has failed to continuously and correctly guide ES, EI, and SI towards favorable development, so the role played by these is unstable.
3. The contribution values of influencing factors calculated using LMDI and RLI models were basically same, and the deviation rate of contribution values was small, remaining within 5%. Using the LMDI model to decompose the factors influencing energy-related carbon emissions in Northwest is scientific and rational.
4. Northwest energy carbon emissions and economic growth show positive decoupling trends, with WD achieved in most years. The favorable decoupling state including WD, EC, and END, account for up to 75%. That's mainly because the Northwest has been actively following the China government's economic guidance to maintain sound economic development in 2000–2020, and meanwhile reduces carbon emissions through developing clean energy, improving energy utilization technology, and developing tertiary industries, which will gradually decouple carbon emissions from economic growth.
5. Overall, G and US hamper decoupling in Northwest, PA reverses, and ES, EI, and SI play an unstable role. G & US is not only the major contributor to carbon emissions, but also to economic growth, so G & US is bound to impede decoupling. On the one hand, PA promotes economic development by driving agglomeration effects in economy through population agglomeration. On the other hand, PA reduces public energy consumption and carbon emissions through population agglomeration, thus PA promotes decoupling. The role played by ES, EI, SI on decoupling needs to be analyzed specifically in the context of annual energy policies, industrial policies and energy utilization technology in Northwest.

### Recommendations

Based on the data derived from calculations, the following recommendations are provided for reducing carbon emissions and achieving the ideal decoupling state in Northwest China.

1. The government needs to leverage both the positive contribution of economic development and urban expansion to social progress and reduce its contribution to carbon emissions. Northwest China should shift the focus of economic development from fossil-energy-dependent industries to third-sector industries that consume less energy. Local governments should publicise and develop its unique natural landscapes such as grasslands, mountains, and icebergs, to actively promote the tourism industry. New built-up areas should be equipped with high-quality and efficient supporting facilities, comprehensive and convenient public service facilities, and low-carbon and comfortable environmental infrastructures.
2. Fully exert population agglomeration effect to inhibit carbon emissions and promote decoupling. The government should formulate a favourable population transfer policy to attract rural populations to urban areas. Linking new built-up areas with the absorption of the agricultural transfer population increases the financial support of incentives for citizenship of the agricultural transfer population.
3. Promote cleaner, low-carbon, and diversified energy structures based on local energy resources. The government should guide local energy companies to focus on exploiting clean energy power generation, building diversified clean energy production bases, diversifying the use of biomass energy, learning about clean and efficient coal mining and utilisation technologies, and enhancing gangue utilisation. The government can levy an energy tax on high-energy-consuming enterprises to force them to increase their use of clean energy and promote the clean energy market penetration rate.
4. Adjust the industry structure and accelerating its upgrade and renewal. The government should rigorously investigate high-energy-consuming, high-emission, and low-yield industries and eliminate backward enterprises. Meanwhile, advanced technology and modern management experience should be introduced into traditional high-energy-consuming industries to implement energy-saving reforms and transform the traditional manufacturing industry into green manufacturing. Finally, favourable policies should be established to attract capital and researchers, and high-tech industries should be introduced to promote the high-end and green transformation of local industries.
5. Improve energy utilisation by focusing on fossil energy efficiency and clean energy utilisation. The government should increase the supervision and diagnosis of high-energy-consuming industries, promote energy-saving and low-carbon technology and equipment in industries, and focus on improving the energy utilisation rate of high-energy-consuming enterprises. Meanwhile, refined and standardised energy utilisation guidelines should be revised, and favourable policies srovided to enterprises that meet the standard energy consumption rate.

## Data Availability

The datasets used and analyzed during the current study are available from the corresponding author on reasonable request.
